# Differential Expression of Immune Defences Is Associated with Specific Host-Parasite Interactions in Insects

**DOI:** 10.1371/journal.pone.0007621

**Published:** 2009-10-27

**Authors:** Carolyn Riddell, Sally Adams, Paul Schmid-Hempel, Eamonn B. Mallon

**Affiliations:** 1 Department of Biology, University of Leicester, Leicester, United Kingdom; 2 Department of Biological Sciences, University of Warwick, Coventry, United Kingdom; 3 Institute of Integrative Biology (IBZ), ETH Zurich, Zurich, Switzerland; University of Zurich, Switzerland

## Abstract

Recent ecological studies in invertebrates show that the outcome of an infection is dependent on the specific pairing of host and parasite. Such specificity contrasts the long-held view that invertebrate innate immunity depends on a broad-spectrum recognition system. An important question is whether this specificity is due to the immune response rather than some other interplay between host and parasite genotypes. By measuring the expression of putative bumblebee homologues of antimicrobial peptides in response to infection by their gut trypanosome *Crithidia bombi*, we demonstrate that expression differences are associated with the specific interactions.

## Introduction

A large number of ecological studies in invertebrates show that the outcome of an infection is dependent on the interaction between the host and pathogen genotypes, and is highly specific [1]. Such specificity contrasts the long-held view that invertebrate innate immunity depends on a broad-spectrum recognition system, only capable of responding very generally to different classes of pathogens [2]. Recent work is now changing this view, as several novel mechanisms for the somatic diversification of immune receptor molecules as well as genetically based polymorphism have been discovered in invertebrates [3]. Down syndrome adhesion molecule (Dscam) in the mosquito, for example, is a hypervariable receptor involved in phagocytosis where the relative frequency of alternatively spliced transcripts might vary in relation to the infecting pathogen. Whilst such mechanistic studies suggest a possible basis of specificity, little work has yet been carried out to directly test whether there is specificity in the interaction between host immune responses and parasite types. Alternatively, specificity might be due to other, non-immune responses associated with host-parasite interactions [4]. Here, we demonstrate that the expression of immune genes coding for important effector molecules varies with the specific combination of host and parasite type.

The interaction of the trypanosomal gut parasite *Crithidia bombi* with its host, the bumblebee, *Bombus terrestris* is highly specific and provides an excellent test case for such questions [5]. In this system, infection success depends on which strain is infecting which colony (representing very different genotypic backgrounds) leading to highly specific assorting of parasite genotypes across different hosts [6]. To address whether the invertebrate innate immune response is specific, we directly measure the bumblebee immune response during a specific interaction with its parasite *C. bombi*. Flagellates such as *Leishmania*, *Trypanosoma sp.* and *Crithidia* develop exclusively in their insect host's gut and do not migrate into the haemolymph. Local immune responses in the gut-epithelium, including antimicrobial peptide (AMP) production [7], [8], [9], are therefore likely to be important in controlling these infections [10]. In the sand fly *Phlebotomus duboscqi*, defensin is induced in the gut epithelia and systemically in the fat-body during *Leishmania major* infection [11]. Similar AMP induction is cited in the insect host's immune response to *Trypanosoma brucei* [12] and *Crithidia sp*. [13].

Based on this literature, we decided to measure the level of AMP gene expression as a signal of potentially differential immune responses. To identify bumblebee homologues of these target genes, we first gathered partial expressed sequence data for the AMPs *defensin 1* and *hymeoptaecin* in *Bombus terrestris* (GenBank accession numbers: FJ839454: *Defensin* and FJ839453: *Hymenoptaecin*) and primers for *abaecin* from *Bombus ignitis* (GenBank accession: AY423049). In a first test, we confirmed that these three AMPs are upregulated upon infection by *C. bombi* (see Supplementary Material S1). Then, to test the specificity of AMP expression, *B.terrestris* workers from four host lines (as defined by colony identity) were naturally infected with one of four *C.bombi* isolates and the bees' expression levels of the three *B.terrestris* AMPs were measured using qPCR.

## Materials and Methods

Experiments were carried out on two commercially reared bumblebee colonies from Koppert Biological Systems U.K. and two colonies from wild caught queens. All parasite isolates used originated from wild queens collected in Spring 2008 in the botanical gardens, University of Leicester. Experiments began when the colonies had a minimum of thirty workers, approximately four weeks old. Between observations, colonies were fed ad libitum with pollen (Percie du sert, France) and 50% diluted glucose/fructose mix (Meliose – Roquette, France). Before and during the experiments colonies were kept at 26°C and 60% humidity in constant red light.

### Infections

To prepare *C. bombi* isolates, faeces was collected from workers of naturally infected colonies, and mixed with 50% diluted Meliose to create a standardized dose of 500 *Crithidia* cells per µl of inoculum. Previous studies had shown that such inocula, prepared from different colonies, are genotypically different [6] and generate specific responses in novel hosts [14]. We infected a sample of workers from each of four bumblebee colonies (representing different host lines) with an inoculum of faeces from each of the four wild infected colonies (mean number of bees +/− Standard Deviation  = 5.4 +/− 0.9, 7 uninfected controls, 93 bees in total). Bees were four days old at the time of infection. After infection bees were kept in colony x strain groups (1–3 individuals depending on day collected) and fed *ad libitum*. 24 hours or 48 hours post infection the bees were sacrificed by freezing in liquid nitrogen. They were then stored at −80°C.

### RNA extraction and cDNA synthesis

Total RNA was extracted from individual homogenised abdomens using Tri-reagent (Sigma-Aldrich, UK). Any residual contaminants were removed from the RNA using the RNeasy mini kit (Qiagen, UK) and manufacturer's RNA clean-up protocol. To remove residual genomic DNA (gDNA), RNA samples were treated with DNase (Sigma-Aldrich, UK). First strand cDNA synthesis was carried out by reverse transcription of 2 µg of total RNA with M-MLV reverse transcriptase (Promega, UK) and oligo dT_23_ primer (1 µg/µl) according to the manufacturer's instructions.

### Quantitative PCR (qPCR) analysis

After synthesis cDNA samples and controls were diluted 10 fold with nuclease-free water. Each qPCR reaction contained 5 µl of dilute cDNA or control, 1x SYBR Green JumpStart Taq ReadyMix (Sigma-Aldrich, UK) and gene specific primers (final concentration of 0.1 µM). For oligonucleotide sequences please see table 1 below. Each sample was tested with the housekeeping gene RPS5 [15] and all 3 AMPs. For design of primers please see supplementary material. Two technical replicates were run per reaction. Reactions for qPCR were prepared using the Corbett robotics machine (Qiagen, UK) and performed on the MJ Research Chromo 4^TM^ (Genetic Research Instrumentation Ltd, Essex, UK) using the following program: 95°c for 5 minutes, followed by 42 cycles of a 30 second 95°C denaturation, 30 second 61°C annealing and 30 second 72°C extension steps.

**Table 1:** Primers used in qPCR.

Gene	Forward primer Sequence	Reverse primer sequence	Tm forward/reverse	Annealing temperature
Bombus RPS5	5′-TCGTCGTAACGAGAAACATCC-3′5′	5′-GAGAAGATTCCACGCGTATTGG-3′	67/66.5°C	60–62°C
Abaecin	5′-ATGAAGGCAGTAATGTTTATTTTC-3′	5′-GGAAAGGTTGGAAACGGTTTAGAT-3′	59/65.8°C	60–62°C
Defensin	5′-AACTGTCTCAGCATGGGCAAAG-3′	5′-AGAGATCCTTGAGTTGGTCTTGC-3′	67.5/65.7°C	60–62°C
Hymenoptaecin	5′-CCTTGTTATCGATGGAAAGAAACC-3-	5′-GTTGATGATAATCGACGTCCAAGG-3′	67.2/65.3°C	60–62°C

C_T_ values were taken at a threshold fluorescence value of 0.02. ΔC_T_ of each sample was calculated by normalising it to the lowest C_T_ value in the control (non-infected) samples in both house keeping genes and AMPs (C_T control_ - C_T sample_). Fold change in expression was calculated with the 2^(Δ−ΔCT)^ approximation method, using the housekeeping *RPS5* as the reference gene. Replicate measurements showed that measurement error was very small (mean 0.6%, range 0.007 to 2.31%). Using fold change instead of absolute values should - at least partially - control for differences in host condition that might affect the overall level of expression of antimicrobial peptides. Furthermore, host condition was additionally controlled as the colonies were kept in exactly the same conditions in the same controlled environment room. They were all fed at the same time from food prepared at the same time. They are never outside the controlled environment. Experiments did not begin till each colony had reached an identical size. Hence, we assume that colony-specific differences in host condition are very unlikely to explain our results.

### Statistical analysis

Fold changes in abaecin and defensin gene expression were box-transformed, and hymenoptaecin zero-skewness log-transformed to fit the data to a normal distribution. Fold data for all three AMPs was first analysed using a MANOVA, and if significant separate ANOVAs was carried out for each AMP. All data analyses were performed using Intercooled STATA 8.2 for Macintosh.

## Results and Discussion

**Figure 1:**
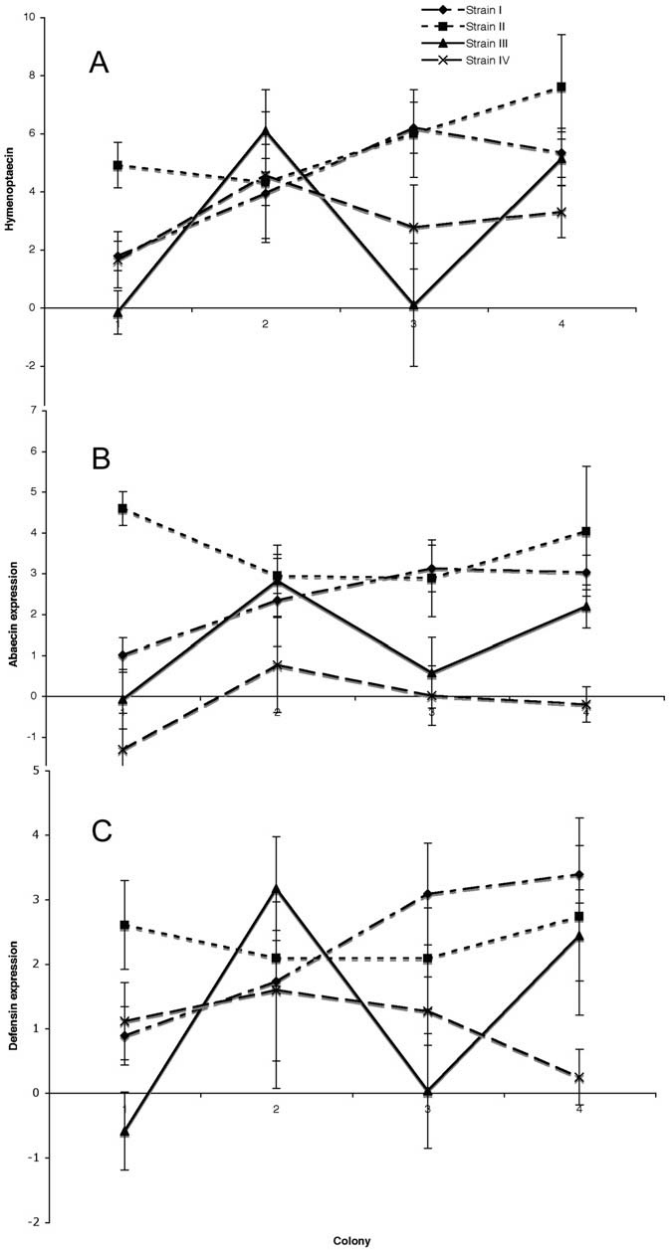
Relative expression of the antimicrobial peptides. Expression levels of (A) hymenoptaecin (values zero skew log transformed as: ln (fold change in hymenoptaecin expression – 0.1952191) normalised to a noninfected control), (B) abaecin (box-cox transformed: Fold change^−1.0302212^/−0.0302212, normalised to a noninfected control) and (C) defensin (box-cox transformed: Fold change^−0.955746^/0.044254, normalised to a noninfected control) across four *B.terrestris* colonies (host lines) in response to four different *Crithidia* isolates (see in-graph legend). Points represent the means and error bars represent the standard errors.

Our results showed a clear main effect of host line on AMP expression (MANOVA with the three AMPs as responses; overall model: *F*_9,155.9_ = 2.28, *P* = 0.02; Wilks' **λ** = 0.7405), and especially for the expression of *hymenoptaecin*: (ANOVA *F*_3,66_ = 5.19; *P* = 0.0028); a main effect of parasite isolate (overall MANOVA: *F*_9,155.9_ = 9.25, *P* = <0.00001; Wilks' **λ** = 0.3530), especially on the expression of *abaecin* (ANOVA: *F*_3,66_ = 13.76; *P* = <0.00001) and *hymenoptaecin* (ANOVA *F*_3,66_ = 4.31; *P* = 0.0078), as well as possible effects of time post-infection on *defensin* expression (measuring gene expression at 24 or 48 hours, overall MANOVA not significant; *defensin* expression: ANOVA *F*_1,66_ = 4.34; *P* = 0.041). The group in which a bee was held during infection had no effect (MANOVA: *F*_60,153_ = 1.19, *P* = 0.2031; Wilks' **λ** = 0.3200). Most importantly and in line with our hypothesis, there is a significant colony*strain interaction effect on AMP expression (MANOVA: *F*_27,187.6_ = 2.30, *P* = 0.0006; Wilks' **λ** = 0.4332), and in particular for two out of three AMPs measured, i.e. *defensin* (ANOVA: *F*_9,66_ = 2.12; *P* = 0.0396), *hymenoptaecin* (ANOVA: *F*_9,66_ = 2.14; *P* = 0.0380), but not for *abaecin* (ANOVA: *F*_9,66_ = 1.6; *P* = 0.1328, see Figure 1). As an example of this interaction, host line 4 shows similar levels of *hymenoptaecin* expression in response to infection by both strain I and III, whereas in host line 3 strain I induces much higher *hymenoptaecin* expression compared to strain III. Hence, the expression of important AMPs varies depending on who is infected by whom. This provides the first experimental evidence that active immune responses are associated with the highly specific interactions observed in ecological studies with invertebrates that have used life-history traits such as survival or fecundity to represent immunity [16], and that these interactions are not solely an artefact of uncontrolled factors [4].

Each of the three AMPs showed similar patterns of expression for each host-parasite pairing (Figure 1). This is to be expected as all three are thought to be controlled by the Imd pathway [17] although this may be more complicated [18]. What we have discovered is specificity in the expression of effectors during the immune response. Further work is required to elucidate the mechanistic basis of this specificity which could be the result of particular receptors, regulatory pathways or a combination of these [5] such as is the case with mechanisms based on Dscam and fibrinogen-related proteins (FREPs), two highly variable protein recognition receptors that bind pathogen-bound epitopes highly specifically and associate with immune tissues [19], [20], [21].

All tested animals became infected, but for practical reasons, it was not possible to simultaneously measure infection intensity and the levels gene expression, as the animal was sampled relatively shortly after infection. Hence, the current study could not show a direct correlation between infection intensity and expression of anti-microbial peptides. However, variation in infection intensity has been observed so universally that it must be taken as given. In fact, the main aim of this study was to test whether, similar to the observation of specific interactions in infection intensities, also variation in the levels of gene expression would show a significant host-parasite interaction term.

Our study now provides evidence that the ecologically important phenomenon of specific host-parasite interactions does indeed have a parallel in the expression profiles of anti-microbial peptides that similarly vary with different host-parasite combinations. We conclude that the general observation of host-parasite specificity in this system has a immunological basis, especially with the differential expression of anti-microbial peptides, which are known effector molecules against trypanosome infections in the gut [7]. Our study emphasises the importance of using natural host-parasite systems when researching specificity of the invertebrate immune defence. Approaching studies of immunity by integration of molecular knowledge into natural host-parasite systems can only serve to enrich our understanding of the higher capabilities and regulation of invertebrate innate immunity.

## Supporting Information

Supplementary Material S1 (0.05 MB DOC)
