# Supplementary material for: Differential Expression of Immune Defences Is Associated with Specific Host-Parasite Interactions in Insects
Source: PLoS One. 2009 Oct 27;4(10):e7621. doi: 10.1371/journal.pone.0007621 (PMC2762600; doi:10.1371/journal.pone.0007621)
Supplement: Supplementary Material S1 — (0.05 MB DOC) [file pone.0007621.s001.doc]

Supplementary material

(1) Identification of Antimicrobial peptide homologues in *Bombus terrestris*.

In order to identify defensin and hymenoptaecin homologues in *Bombus terrestris* we looked for regions of conservation in these genes in other Hymenoptera and other insects.

Abaecin, defensin, hymenoptaecin and RPS5 genes were identified in honeybees or bumblebees (abaecin: *Bombus ignitus* AY423049.1; defensin: *B.ignitus* AY23050; hymenoptaecin: *B.ignitius* EU411043; RPS5 *Apis mellifera* XM_393226.3) and used as the subject in searches of the translated nucleotide NCBI database ([http://www.ncbinlm.nih.gov](http://www.ncbi.nlm.nih.gov/)).

For each gene, two to three sequences (defensin: *A.mellifera* AY333923 & NM_001011616; hymenoptaecin: *B.ignitus* EU411044 & *A.mellifera* NM_001011615; RPS5 *Nasonia vitripennis* XM_001599025.1 & *Diaphorina citri* DQ673389.1) showing the highest degree of homology were aligned using the CLC sequence viewer programme (CLC version 4.6.1. for Macintosh) to identify conserved regions within the sequence. Primers were then designed within these regions of conservation using the perfect-primer software (Invitrogen, UK) and used to amplify sequences from *Bombus terrestris* cDNA from infected bees.

Abaecin was designed slightly differently since few abaecin sequences were available in the NCBI database at the time and homologues in *A.mellifera* did not have adequate sequence conservation to *B.ignitus* to design primers to. In addition, the sole *B.ignitus* sequence available was 177bp long, making it difficult to obtain an initial *B.terrestris* sequence on which to design qPCR primers. Instead, qPCR primers were designed on the *B.ignitus* sequence (GenBank accession AY423049.1), using a previously published forward primer for abaecin (Choi et al. 2008) and a reverse primer designed by eye, and checked using the Sigma DNA calculator ([www.**sigma**-genosys.com/calc/**DNA**Calc.asp](http://www.sigma-genosys.com/calc/DNACalc.asp)) to target an 88bp fragment. These primers were run in an RT-PCR to check for a single product as below but using an annealing temperature of 60°C. Since designing these primers, a study by Xu *et al.* (2009) has identified multiple copies of the abaecin transcript in the Asiatic honeybee *Apis cerana* that show high homology and sequence conservation (90% nucleotide homology) with the *B.ignitus* sequence used to design our primers.

Primer sets were tested using ReadyMix Taq PCR mix with MgCl_2_ (Sigma, UK) and 0.5μl of *B.terrestris* cDNA. To optimise the reaction and produce a single amplicon, primers were run in a PCR on a 96 well Biometra T1 Thermal Cycler using the following cycles: 94°C for 3 minutes then 30-34 cycles of 30 second 94°C denaturation, 30 second 50-55°C annealing step and 1 minute 72°C extension step. PCR reactions were run on a gel, and recovered amplicons were sequenced (John Innes Centre, Norwich, UK). The identity of these primary amplicons was checked using BLASTn, and all showed high homology to the desired antimicrobial genes (>70% homology) in *Apis sp.*, *Bombus sp.* and *N.vitripennis.* RPS5 was highly homologous to many insect RPS5 genes, including *A.mellifera, Bombus sp.* and species of butterfly (*Aporia crataegi*) and wasp (*Lysiphlebus testaceipes*) with >70% nucleotide sequence homology. All *B.terrestris* sequences were submitted to the GenBank database. See paper for AMP accession numbers. RPS5 accession: (FJ931041). Primers were then designed within the Bombus sequence to produce an amplicon of around 100bp, suitable for qPCR analysis. Primers were synthesised by Sigma-Aldrich, UK.

(2) Infection with *C. bombi* leads to changes in AMP expression.

Three bees were infected with a mixed-strain *C.bombi* inoculum as detailed above and sacrificed at 12 h post-infection. Uninfected controls were collected from a separate colony to provide a baseline for each AMP’s expression. cDNA was synthesised from the total RNA of individual bees and analysed with qPCR using the same conditions and house-keeping gene RPS5 as described for cross-infection experiment. Infected and non-infected expression was transformed to fit the data to a normal distribution, and compared using a standard one-way ANOVA. Expression of abaecin, defensin and hymenoptaecin was significantly up-regulated after 12 h (abaecin F(1,4) = 21.76, P = 0.0096; defensin F(1,4) = 13.45, P = 0.0214; hymenoptaecin F(1,3) = 36.83, P = 0.0090).

Y. Choi et al., Comparative Biochemistry and Physiology Part B: Biochemistry and Molecular Biology **150**, 141 (June 1, 2008).

P. Xu, M. Shi, X. Chen, F. Ausubel, *PLoS ONE* **4**, e4239 (2009).
